# Supplementary material for: Quantum Dot-Induced Blue Shift of Surface Plasmon Spectroscopy
Source: Nanomaterials (Basel). 2022 Jun 16;12(12):2076. doi: 10.3390/nano12122076 (PMC9230993; doi:10.3390/nano12122076)
Supplement: Supplementary file 1 [file nanomaterials-12-02076-s001.zip › nanomaterials-1750967-supplementary.pdf]

nanomaterials

Supplementary Materials

Quantum Dot-Induced Blue Shift of Surface Plasmon Spectroscopy

Than Thi Nguyen [1], Vien Thi Tran [1], Joo Seon Seok [1], Jun-Ho Lee [2] and Heongkyu Ju [1,*]

[1] Department of Physics, Gachon University, Seongnam 13120, Korea; nguyenthan1093@gmail.com (T.T.N.); tranvien04@gmail.com (V.T.T.); wntjs0807@gmail.com (J.S.S.)
[2] Laser& Opto-Electronics Team, Korea Electronics Technology Institute (KETI), Seongnam 13509, Korea; junholee@keti.re.kr
* Correspondence: batu@gachon.ac.kr

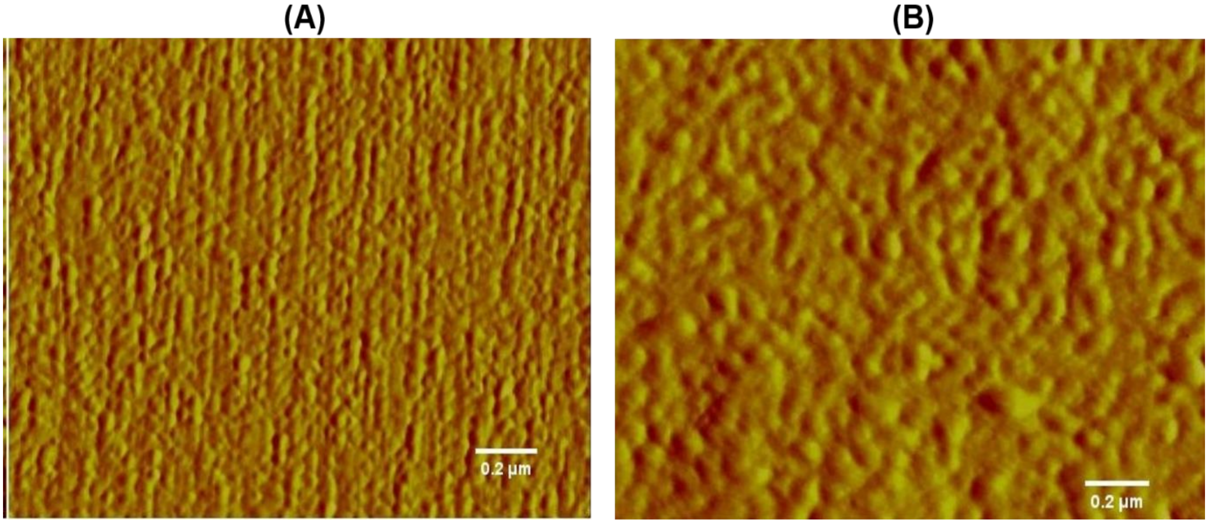

Figure S1. The atomic force Microscope (Veeco Metrology system, model No. 920-006-101) image of the surface. (**A**) 2nm Au−50 nm Ag (bimetallic film)/fiber silica core (**B**) QDs/2nm Au−50 nm Ag (bimetallic film)/fiber silica core. A Si_3N_4 cantilever tip of its radius <10nm (RTESP, Bruker, Santa Barbara, CA,USA) is used.

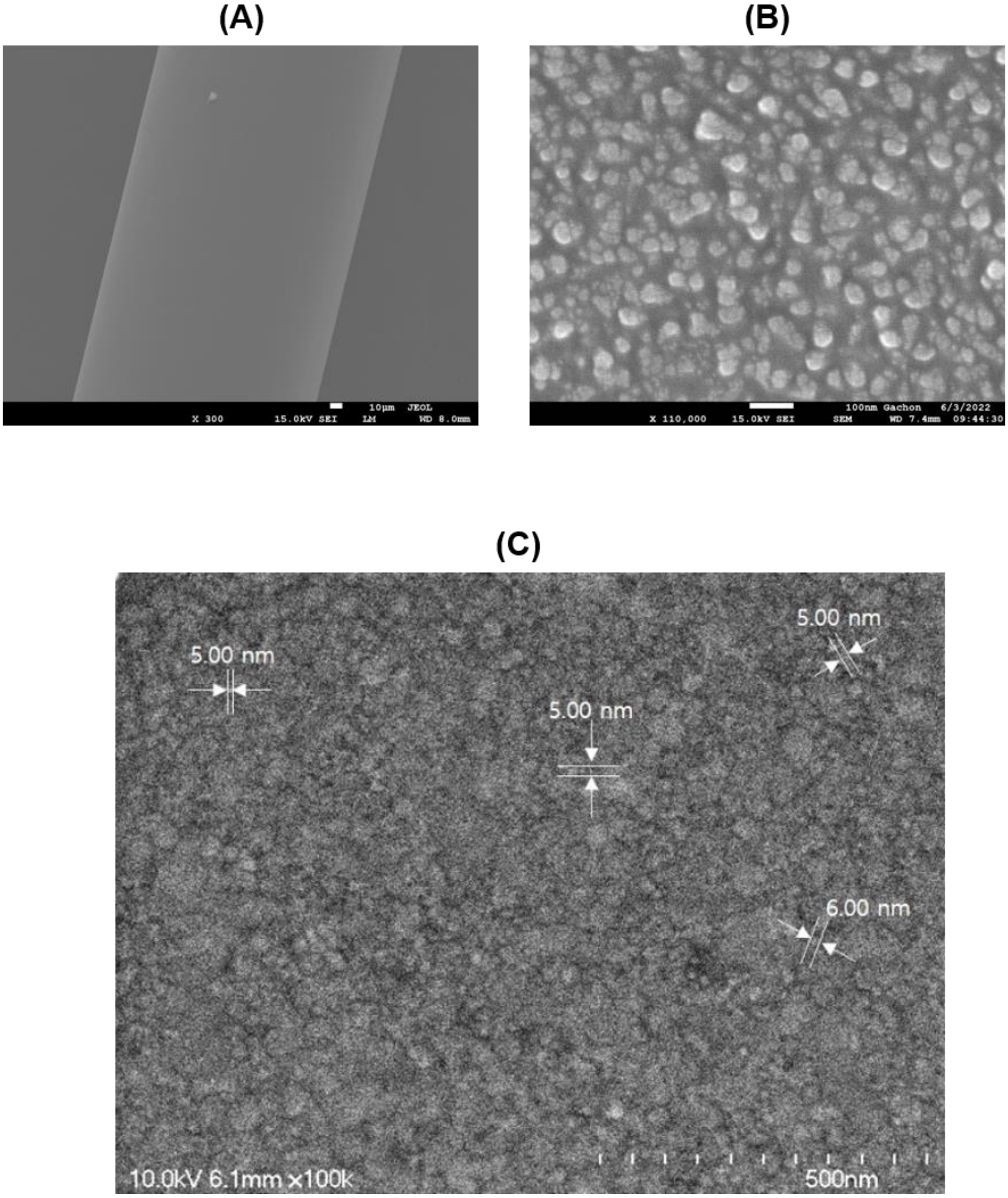

Figure S2. Scanning electron microscope (SEM) images. (**A**) SEM image (JSM-7500F, JEOL) of silica core surface of the clad-free optical fiber. (**B**) SEM image (JSM-7500F, JEOL) of surface of QDs/2nm Au−50 nm Ag (bimetallic film)/silica core surface of the clad-free fiber. (**C**) SEM image (S4700, Hitachi) of QDs/2nm Au−50 nm Ag (bimetallic film)/glass substrate. .
